# Tolfenamic acid inhibits ROS-generating oxidase Nox1-regulated p53 activity in intrastriatal injection of malonic acid rats

**DOI:** 10.1186/s12576-022-00842-4

**Published:** 2022-07-18

**Authors:** Xin Yang, Heling Zhang, Tong Qu, Yi Wang, Yongxian Zhong, Yuchen Yan, Xuefei Ji, Tiayan Chi, Peng Liu, Libo Zou

**Affiliations:** 1 Department of Pharmaceutics, Shenyang Pharmaceutical University, 103 Wenhua Road, Shenhe, Shenyang, 110016 Liaoning China; 2 Department of Pharmacology, Shenyang Pharmaceutical University, 103 Wenhua Road, Shenhe, Shenyang, 110016 Liaoning China

**Keywords:** Tolfenamic acid, Malonic acid, ROS, Nox1, p53

## Abstract

It has been reported that wild-type p53-induced gene 1 (Wig1), which is downstream of p53, regulates the expression of mutant huntingtin protein (mHtt) in Huntington’s disease (HD) patients and transgenic mouse brains. Intrastriatal injection of malonic acid in rats is often used as a model to study the pathological changes of Huntington’s disease, and this model has the advantages of a fast preparation and low cost. Therefore, in this study, we used intrastriatal injections of 6 μM malonic acid in rats to evaluate the effect of tolfenamic acid on motor and cognitive deficits and the effect of 6 mg/kg and 32 mg/kg tolfenamic acid on p53 and its downstream targets, such as Wig1. The results showed that 32 mg/kg tolfenamic acid attenuated motor and spatial memory dysfunction, prevented Nox1-mediated reactive oxygen species (ROS) production, and downregulated the activity of p53 by increasing the phosphorylation level at the Ser378 site and decreasing the acetylation level at the Lys382 site. Tolfenamic acid reduced mouse double minute 2 (Mdm2), phosphatase and tensin homologue (Pten), P53-upregulated modulator of apoptosis (Puma) and Bcl2-associated X (Bax) at the mRNA level to inhibit apoptosis and downregulated sestrin 2 (Sesn2) and hypoxia inducible factor 1, alpha subunit (Hif-1α) mRNA levels to exert antioxidative stress effects. In addition, 32 mg/kg tolfenamic acid played a role in neuroprotection by decreasing the terminal deoxynucleotidyl transferase-mediated dUTP nick end labelling (TUNEL)-positive cell numbers. However, there was no difference in the Wig mRNA level among all groups, and tolfenamic acid could not decrease the protein level of Wig1. In conclusion, tolfenamic acid inhibited the ROS-generating oxidase Nox1-regulated p53 activity and attenuated motor and spatial memory deficits in malonic acid-injected rats.

## Introduction

Huntington’s disease (HD) is an autosomal-dominant neurodegenerative disease caused by abnormal formation of mutant huntingtin protein (mHtt). Tolfenamic acid, a nonsteroidal anti-inflammatory drug, shows anti-inflammatory effects through cyclooxygenase inhibition mechanisms [1]. It regulates the protein kinase C (PKC)/protein kinase B (Akt)/IkappaB kinase (IKK)/noncanonical nuclear factor-κB (NF-κB) signalling pathways to improve anti-inflammatory activity and reduce toxicity in mice [2]. Tolfenamic acid also shows antioxidative effects. It increases the mRNA levels of nuclear factor erythroid 2-related factor 2 (Nrf2), and its vital target genes NAD(P)H quinine oxidoreductase 1 (NQO1) and haem oxygenase 1 (HO1) regulate the stabilization of glutathione (GSH) and oxidized GSH [3]. Tolfenamic acid decreases the production of superoxide (O2−) in normal human polymorphonuclear leukocytes [4].

Our previous study found that tolfenamic acid could prevent motor and memory dysfunction in R6/1 HD transgenic mice. Its mechanism may be related to downregulating specificity protein 1 (Sp1) expression, enhancing autophagy levels, and further promoting mHtt clearance and antioxidant stress injury [3]. In a nontransgenic model, we found that pretreatment with tolfenamic acid can attenuate 3-nitropropionic acid-induced muscular weakness in the forelimb and prevent mitochondrial dysfunction in the brains of mice [5]. Mitochondrial II complex (succinate dehydrogenase, SDH) activity is decreased in the brains of HD patients [6]. Both malonic acid and 3-nitropropionic acid are mitochondrial toxins that can inhibit SDH activity and are commonly used for preparing Huntington models [7, 8].

Nicotinamide adenine dinucleotide 3-phosphate (NADPH) oxidase 1 (Nox1) is an isoform of the Nox family that plays a role in generating superoxide and reactive oxygen species (ROS) in addition to mitochondria. In the CNS, Nox1 is upregulated in ischaemic and traumatic brain injury [9] and promotes α-synuclein expression [10]. Delivery of miRNA against Nox1 to the ventral tegmental area restores cortistatin-induced depressive-like behaviours [11]. Meijles et al. reported that Nox1-dependent generation of ROS increases the activity of the transcription factor p53, and Nox1 inhibition decreases p53 nuclear localization [12]. As a tumour suppressor, p53 regulates apoptosis. Under normal physiological conditions, intracellular p53 was maintained at a low level. When cells are stressed, the expression and activity of p53 increase, resulting in cell cycle arrest, abnormal cell proliferation and apoptosis [13]. It has been reported that the nuclear expression and transcriptional activity of p53 are upregulated in HD transgenic mice and patient brains, and knockout of p53 prevents neuronal cell injury and behavioural deficits in mHtt transgenic mice [14]. In the brains of HD patients and HD transgenic model mice, wild-type p53-induced gene 1 (Wig1), which is downstream of p53, preferentially binds to mHtt mRNA, further regulating the expression of mHtt [15]. Inhibiting Wig1 can significantly decrease the cytotoxicity of mHtt and slow its deposition [16]. Based on these previous studies, we hypothesized that p53 and its related pathways may be drug targets for HD.

The present study aimed to examine whether tolfenamic acid can inhibit ROS-generating oxidase Nox1-regulated p53 and its related pathways to prevent malonic acid-induced behavioural dysfunction and neurotoxicity. This study represented an extension of our previous reports [3, 5], with the effect of tolfenamic acid on Nox1-mediated ROS production, and it is also innovative in that it evaluates the effect of tolfenamic acid on the p53 pathway.

## Materials and methods

### Materials

Tolfenamic acid (ab142953, purity > 98%) was purchased from Abcam (USA). Malonic acid (M1296, purity: 99%) and Avertin (T48402, purity: 97%) were purchased from Sigma–Aldrich (USA). Mouse anti-p53 (ab26), p53 Ser378 (ab372), rabbit anti-Nox1 (ab131088), p53 Lys370 (ab183544), p53 Lys382 (ab75754), and NeuN (ab177487) were purchased from Abcam (USA). Rabbit anti-Wig1 (ZMAT3, 10504-1-AP) was purchased from ProteinTech (China). Mouse anti-β-actin, anti-mouse IgG and anti-rabbit IgG were purchased from Santa Cruz (USA).

### Animals

Male Wistar rats (220–250 g) were purchased from LiaoNingChangSheng Biotechnology Co., Ltd. (China). The rats were maintained on a 12 h light/dark cycle. Food and water were provided ad libitum. All animal studies were performed in strict accordance with the P.R. China legislation on the use and care of laboratory animals and with the guidelines established by the Institute for Experimental Animals at Shenyang Pharmaceutical University.

### Bilateral intrastriatal injection of the malonic acid model and tolfenamic acid treatment

The rats were anaesthetized by i.p. 2.5% Avertin. Malonic acid (MA) was injected slowly by a microlitre syringe in a volume of 4 μL of PBS (containing 6 μM malonic acid) at the following coordinates: + 1.7 mm anterior to the bregma, ± 2.7 mm lateral to the sagittal suture and − 4.8 mm ventral [17]. Sham group rats were injected with the same volume of PBS.

**Fig. 1:**
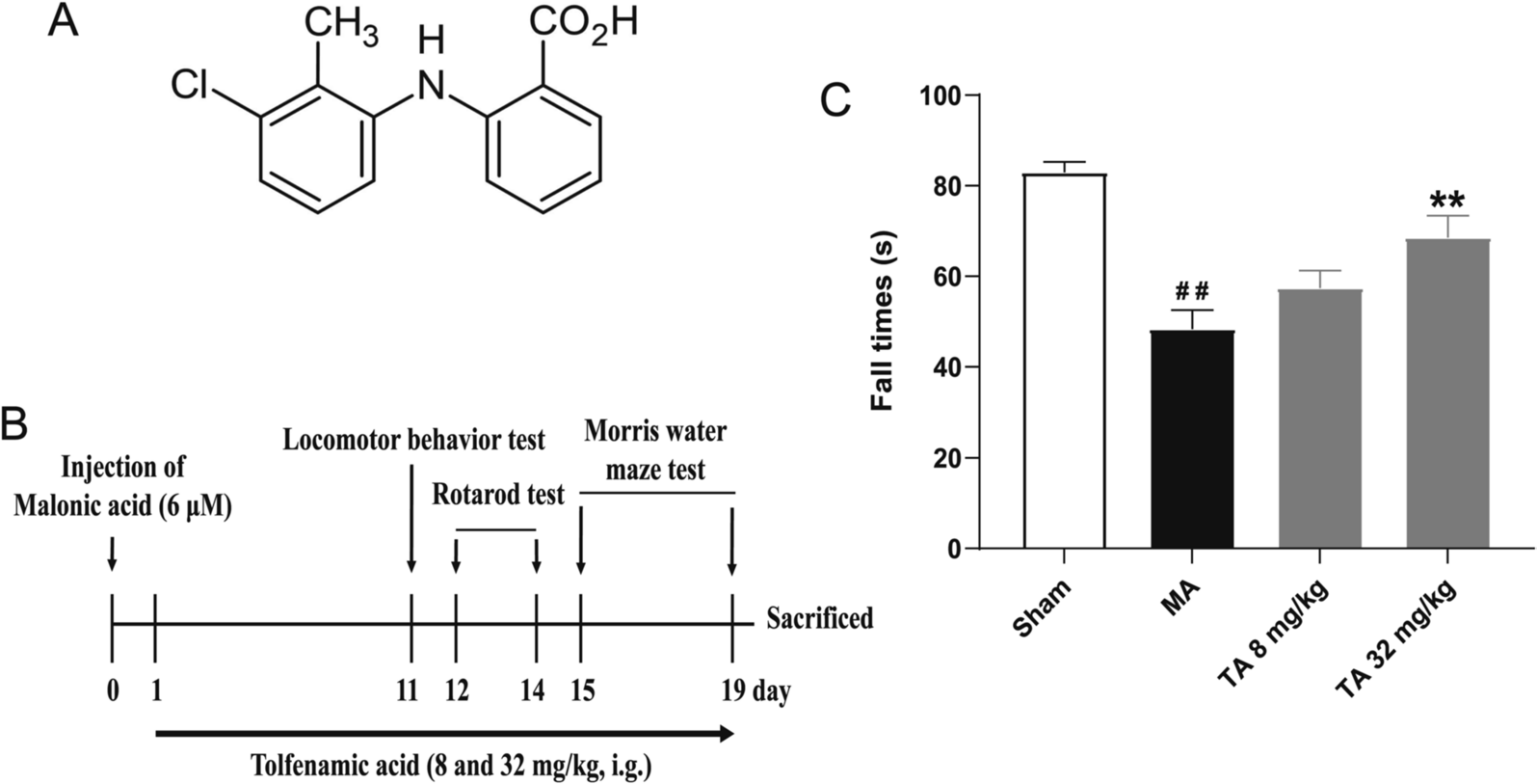
Effect of tolfenamic acid on motor deficits in malonic acid (MA)-injected rats. **A** Chemical structure of tolfenamic acid. **B** Protocol for the experiment. **C** MA group rats showed a decrease in the fall latency time in the rotarod test compared to that of sham group rats. Tolfenamic acid increased the fall latency time. All of the results are expressed as the means ± SEMs. *n* = 10; ##*p* < 0.01 vs. sham; **p < 0.01 vs. MA

The rats were divided into four groups of 10 animals each: sham group, MA group, MA + tolfenamic acid 8 mg/kg (30.65 μM/kg) group and MA + tolfenamic acid 32 mg/kg (122.61 μM/kg) group. The rats were administered tolfenamic acid by gavage. The experimental schedule is summarized in Fig. 1B. After the Morris water maze test, the rats were decapitated under ether anaesthesia, and four brains from each group were used for TUNEL staining. The left striatum from the other rats was used for western blotting and PCR. The right striatum from the other rats was used to measure oxidative stress. The doses of tolfenamic acid were based on the conversion of a regular dose for mice in our previous study [3].

### Locomotor behaviour test

The experimental equipment for the locomotor behaviour test was a PVC enclosed chamber (length, width and height were 45 cm). The rats could explore the chamber for 8 min. The exploration distance, exploration time, movement number, and nonexploration time were recorded.

### Rotarod test

The rotarod test was performed as previously described [3]. Briefly, the rats were given a prior training session for 2 days before the test to acclimatize them to the rotarod equipment (Shanghai Xinruan, China). When performing the test, three separate trials began at 25 rpm over a period of 90 s. The latency to fall values were recorded, and the average time of fall was used for the comparative analyses.

### Morris water maze test

The equipment is a circular pool (150 cm diameter, 50 cm high) with 25 ± 2 ℃ and 30 cm-deep water. In the fourth quadrant, there was a security platform, which was 1 cm below the water. In the training session, the rat swam in the pool for 90 s to find the platform twice a day. If the rat could not find the platform, it was placed on the platform for 20 s. On the fifth day, the platform was removed, and the rat swam for 90 s. The escape latency (time spent in the fourth quadrant), swimming distance, and swimming speed were recorded, as described in our previous report [18].

### Estimation of oxidative stress

Malondialdehyde (MDA), nitric oxide (NO), superoxide dismutase (SOD), catalase (CAT), and H_2_O_2_ levels were measured from striatum extracts prepared in cell lysis buffer using commercially available assay kits (Nanjing Jiancheng Bioengineering Institute, China) according to the manufacturer’s protocol. According to the kit instructions, the content of reduced glutathione = total glutathione−2 × oxidized glutathione.

### HE staining

The brains were fixed in 4% formaldehyde solution (pH 7.0) for at least 24 h. The tissues were processed routinely for paraffin embedding and cut into 5 μm-thick sections. Paraffin sections were stained with haematoxylin solution and alcohol eosin solution. After staining, the sections were dehydrated in anhydrous ethanol and treated in xylene. Then, the sections were dipped with resinous medium and mounted with cover glass for observation.

### TUNEL staining

Apoptotic cells were detected in 5 μm-thick sections with an In Situ Cell Apoptosis Detection Kit I (POD) (Boster, Wuhan, China). The procedures were performed according to the protocol of the kit. Positive cells were identified and counted.

### Immunohistochemical staining

Briefly, brain sections were incubated with Nox1 (1:100) and NeuN (1:200) antibodies at 4 °C overnight. Then, the sections were incubated with biotin-labelled secondary antibody at 37 °C for 30 min. The sections were treated with avidin–biotin enzyme reagent and visualized using a DAB kit (Boster, Wuhan, China). The positive area of each section was quantified using ImageJ software.

### Western blotting

Forty micrograms of protein was separated on 8–12% gradient SDS–PAGE gels and electrophoretically transferred to 0.45-μm PVDF membranes. The membranes were blocked with 5% skim milk for 2 h and then incubated with primary antibodies against p53 (1:1000), p53 Ser378 (1:500), p53 Lys370 (1:500), p53 Lys382 (1:500), Nox1 (1:500), Wig1 (1:1000) and β-actin (1:1000) overnight at 4 °C. Membranes were incubated with secondary antibodies (1:10,000) for 2 h. Immunoreactive bands were visualized using an ECL kit.

**Table 1:** Sequences of primers used

Gene	Type	Primer sequence (5′–3′)
p53:	Forward	GCCATCTACAAGAAGTCACAA
	Reverse	GCCTGTCGTCCAGATACTC
Bax	Forward	TAGCAAACTGGTGCTCAAGG
	Reverse	GGGTCCCGAAGTAGGAAAGG
Puma	Forward	TGTCACCAGCCCAGCAGCAC
	Reverse	TTGAGGTCGTCCGCCATCC
Wig1	Forward	CCATGTGCGTTACTCCAAG
	Reverse	TTCTCCATCTCGCTCCTGT
Sesn2	Forward	TGTTTGGCTGTGGGATACTTC
	Reverse	CCGAGTTGTTCAATGGGTCT
Mdm2	Forward	AGGTCTATCGGGTCACAGT
	Reverse	GCCAGTTCTCACGAAGGGT
Hif-1α	Forward	CTACTATGTCGCTTTCTTGG
	Reverse	GTTTCTGCTGCCTTGTATGG
Cx3cl1	Forward	AGCCTCAGAGCACTGGAAT
	Reverse	GGACGCTTGAGTAGATAGGG
Pten	Forward	AGACCATAACCCACCACAGC
	Reverse	TACACCAGTCCGTCCTTTCC
β-Actin	Forward	GGAGATTACTGCCCTGGCTCCTAGC
	Reverse	GGCCGGACTCATCGTACTCCTGCTT

### Quantitative real-time PCR (RT-PCR)

Total RNA was extracted from the striatum with TRIzol (BioTeke, China). RNA (2.0 μg) was reverse-transcribed using a cDNA synthesis system and then amplified with SYBR GREEN Master Mix (Solarbio, China) on an Exicycler 96 Real-Time PCR Detection System (BIONEER, Korea). The relative mRNA levels were calculated using the standard 2^−ΔΔCt^ relative quantification method. The primers for rat were synthesized by Genscript Biotech Corporation (China), and the sequences were as follows in Table 1.

### Statistical analysis

The data were analysed using SPSS 21.0. The statistical significance was determined using one-way or two-way ANOVA followed by Fisher’s LSD multiple comparisons test with homogeneity of variance or Dunnett’s T3 test with heterogeneity of variance. Kolmogorov–Smirnov test was used to evaluate the distribution of our data. If the data was not normal distribution, we used Kruskal–Wallis test to analyse. Experimental data are presented as the means ± SEMs. A value of *p* < 0.05 indicates statistical significance.

## Results

### Tolfenamic acid prevented motor and spatial memory dysfunction in malonic acid-injected rats

The neurobehavioural function of rats was evaluated by the locomotor behaviour test. The rats were allowed to explore in a PVC enclosed chamber for 8 min without any disturbance, and then the exploration distance, movement speed, and exploration time were recorded to represent locomotor activity. Locomotor activity was significantly decreased after malonic acid injection compared with that of the sham group rats (exploration distance, movement speed, *p* < 0.01; nonexploration time, exploration time, *p* < 0.05, Table 2). Tolfenamic acid (32 mg/kg) prevented the decrease in locomotor test (exploration distance, movement speed, *p* < 0.05, Table 2). There was no difference in the movement number among all groups. The motor coordination of rats was evaluated by the rotarod test. Malonic acid injection significantly decreased the average time of fall values in the rotarod test (*p* < 0.01, Fig. 1C). Tolfenamic acid (32 mg/kg) significantly attenuated the decrease in latency to fall values (*p* < 0.01, Fig. 1C).

**Table 2:** Effect of tolfenamic acid on locomotor behaviour in malonic acid injected rats

Group	Exploration distance (mm)	Movement speed (mm/s)	Movement number	Non-exploration time (s)	Exploration time (s)
Sham	31933.13 ± 2173.29	66.53 ± 4.53	76.60 ± 4.53	73.94 ± 4.58	406.06 ± 4.58
MA	21916.30 ± 1425.99^c^	45.66 ± 2.97^c^	78.70 ± 4.71	103.39 ± 12.92^b^	376.61 ± 12.92^b^
TA 8 mg/kg	23956.65 ± 1532.72	49.91 ± 3.19	76.00 ± 2.94	119.65 ± 13.17	360.35 ± 13.17
TA 32 mg/kg	28304.47 ± 1578.96^a^	58.97 ± 3.29^a^	64.80 ± 4.46	77.16 ± 7.49	402.84 ± 7.49

All of the results are expressed as the means ± SEM. *n* = 10

^a^*p* < 0.05 vs. MA group

^b^*p* < 0.05

^c^*p* < 0.01 vs. sham group

**Fig. 2:**
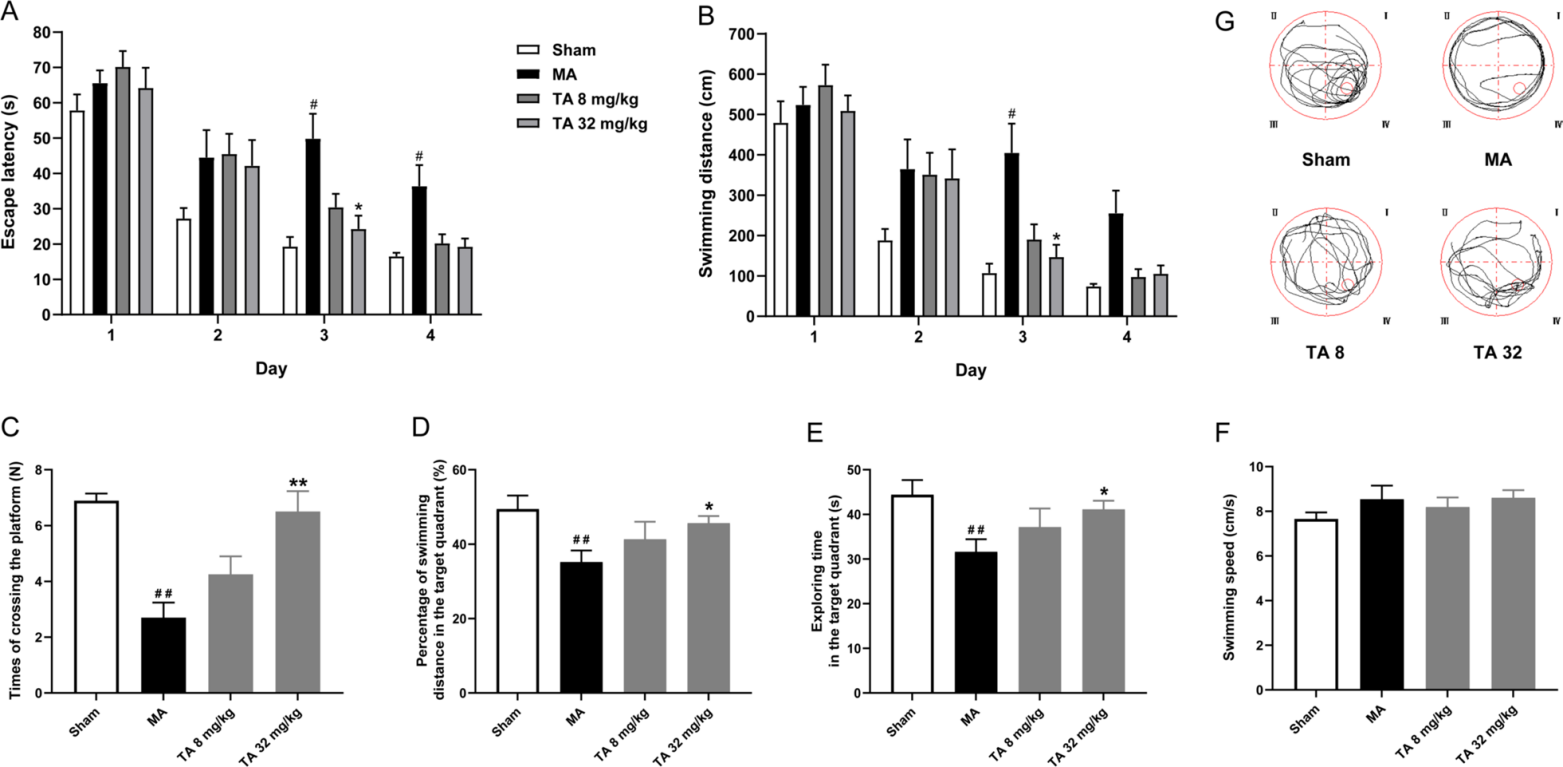
Effects of tolfenamic acid on spatial memory deficits in malonic acid-injected rats. MA group rats exhibited a longer swimming time (**A**) and swimming distance (**B**) to reach the platform during the training period in the Morris water maze test. MA group rats showed a decrease in the swimming time (**C**), swimming distance (**D**) and number of times crossing the platform (**E**) in the target quadrant in the probe test. These effects were reversed by tolfenamic acid. No significant differences in swim speed were observed among all groups (**F**). The results are expressed as the means ± SEMs. *n* = 10; #*p* < 0.05, ##*p* < 0.01 vs. sham; *p < 0.05, **p < 0.01 vs. MA

The Morris water maze test was used to evaluate malonic acid-induced spatial memory impairment in rats. In the training section, malonic acid-injected rats showed a longer swimming time and distance to find the platform than those of the sham group rats (Fig. 2A, B). In the probe test, the exploration time, the percentage of the exploration distance spent in the target quadrant and the number of times the model rats crossed the platform were 31.64 ± 2.79 s, 35.19 ± 3.10% and 2.70 ± 0.54, respectively, which were shorter than those of the sham group rats (*p* < 0.01, Fig. 2C–E). Tolfenamic acid group rats spent more time (41.14 ± 1.91 s), travelled a longer distance (45.67 ± 1.89%) in the target quadrant, and crossed the platform more times (6.50 ± 0.73) than did model group rats (*p* < 0.05, Fig. 2C–E). There were no significant differences in the swim speed among all groups (*p* > 0.05, Fig. 2F). All of the above results indicated that tolfenamic acid could attenuate locomotor activity, motor function and spatial memory deficits in malonic acid-injected rats.

### Tolfenamic acid prevented Nox1-mediated oxidative stress in the striatum of malonic acid-injected rats

**Fig. 3:**
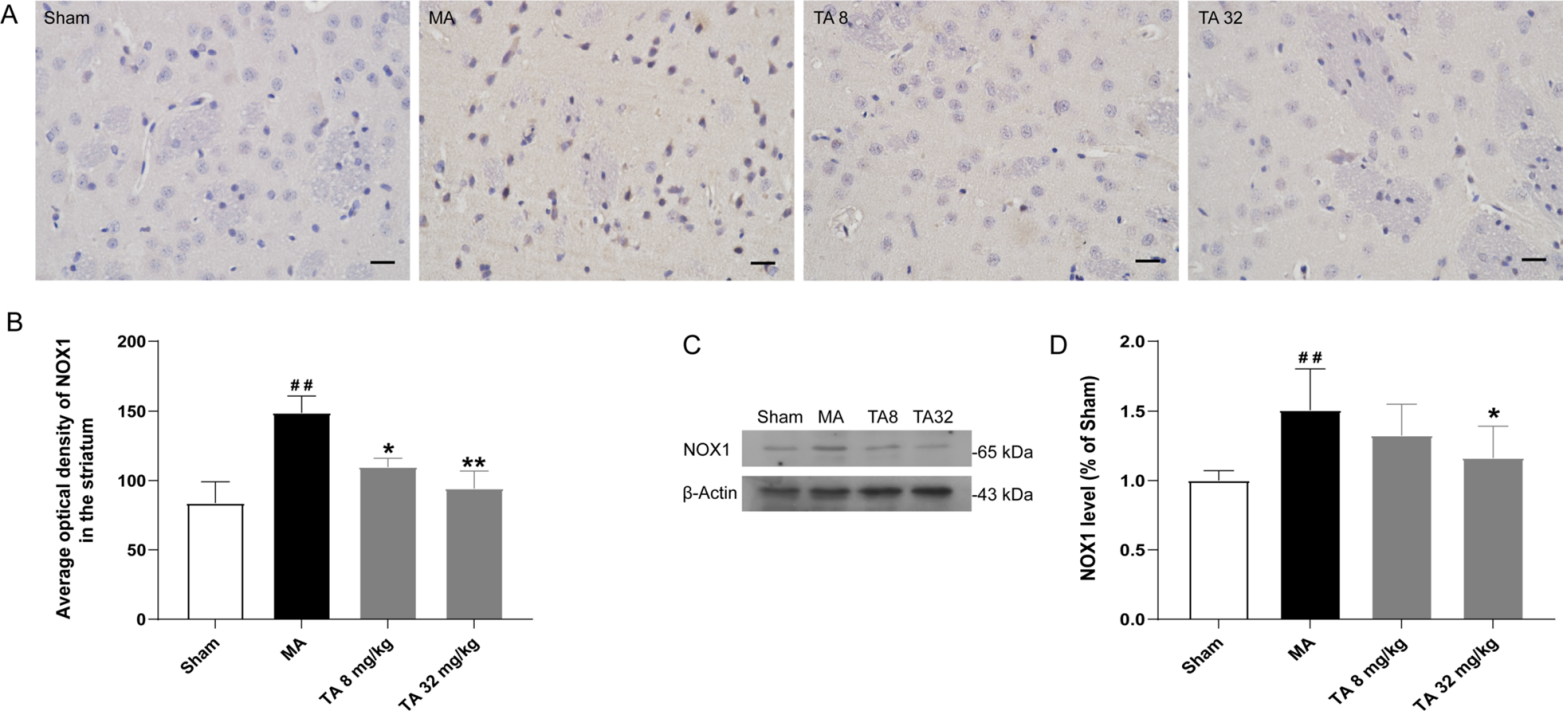
Tolfenamic acid significantly decreased the expression of Nox1 in the striatum of malonic acid-injected rats. Immunohistochemical staining of Nox1 revealed increased Nox1 expression in the striatum of the malonic acid-injected rats and a significant decrease after tolfenamic acid treatment (**A**, **B**). Similar results were observed in the western blot analysis (**C**, **D**). All results are expressed as the mean ± SEM. *n* = 4. Scale bars = 250 μm. ##*p* < 0.01 vs. sham; *p < 0.05, **p < 0.01 vs. MA

Nox1 plays an important role in generating superoxide and ROS. Immunohistochemical staining of Nox1 revealed increased Nox1 expression in the striatum of the malonic acid-injected rats and a significant decrease after tolfenamic acid treatment (p < 0.05, Fig. 3A, B). Similar results were observed in the western blot analysis (Fig. 3C, D). Intrastriatal malonic acid significantly increased the MDA, NO and H_2_O_2_ contents and decreased CAT and SOD enzyme activities in the striatum compared with those of the sham group. Tolfenamic acid (32 mg/kg) significantly decreased lipid peroxidation levels from 16.37 ± 1.37 to 10.49 ± 1.21 nmol/mg protein, the NO content from 1.43 ± 0.07 to 0.54 ± 0.06 μmol/mg protein, and the H_2_O_2_ content from 15.60 ± 1.50 to 9.77 ± 1.71 μmol/mg protein and restored SOD enzyme activity from 37.04 ± 2.28 to 48.37 ± 1.97 U/mg protein, while CAT enzyme activity ranged from 8.89 ± 1.13 to 14.32 ± 1.18 U/mg protein (Table 3). All of the above results indicated that tolfenamic acid could prevent Nox1-mediated oxidative stress in malonic acid-injected rats.

**Table 3:** Effect of tolfenamic acid on oxidative damage (lipid peroxidation, nitric oxide, superoxide dismutase and catalase levels) in the striatum of malonic acid injected rats

Group	MDA (nmol/mg protein)	NO (μmol/mg protein)	H_2_O_2_ (μmol/mg protein)	SOD (U/mg protein)	CAT (U/mg protein)
Sham	9.49 ± 0.88	0.54 ± 0.04	9.05 ± 1.24	54.66 ± 5.11	15.53 ± 1.71
MA	16.37 ± 1.37^c^	1.43 ± 0.07^c^	15.60 ± 1.50^c^	37.04 ± 2.28	8.89 ± 1.13^c^
TA 8 mg/kg	13.98 ± 1.20	1.17 ± 0.11^a^	13.70 ± 1.46	41.63 ± 2.08	11.77 ± 1.67
TA 32 mg/kg	10.49 ± 1.21^b^	0.54 ± 0.06^b^	9.77 ± 1.71^a^	48.37 ± 1.97^a^	14.32 ± 1.18^a^

All of the results are expressed as the means ± SEM. *n* = 5

^a^*p* < 0.05

^b^*p* < 0.01 vs. MA group

^c^*p* < 0.01 vs. sham group

### Tolfenamic acid decreased the activity of p53 in the striatum of malonic acid-injected rats

**Fig. 4:**
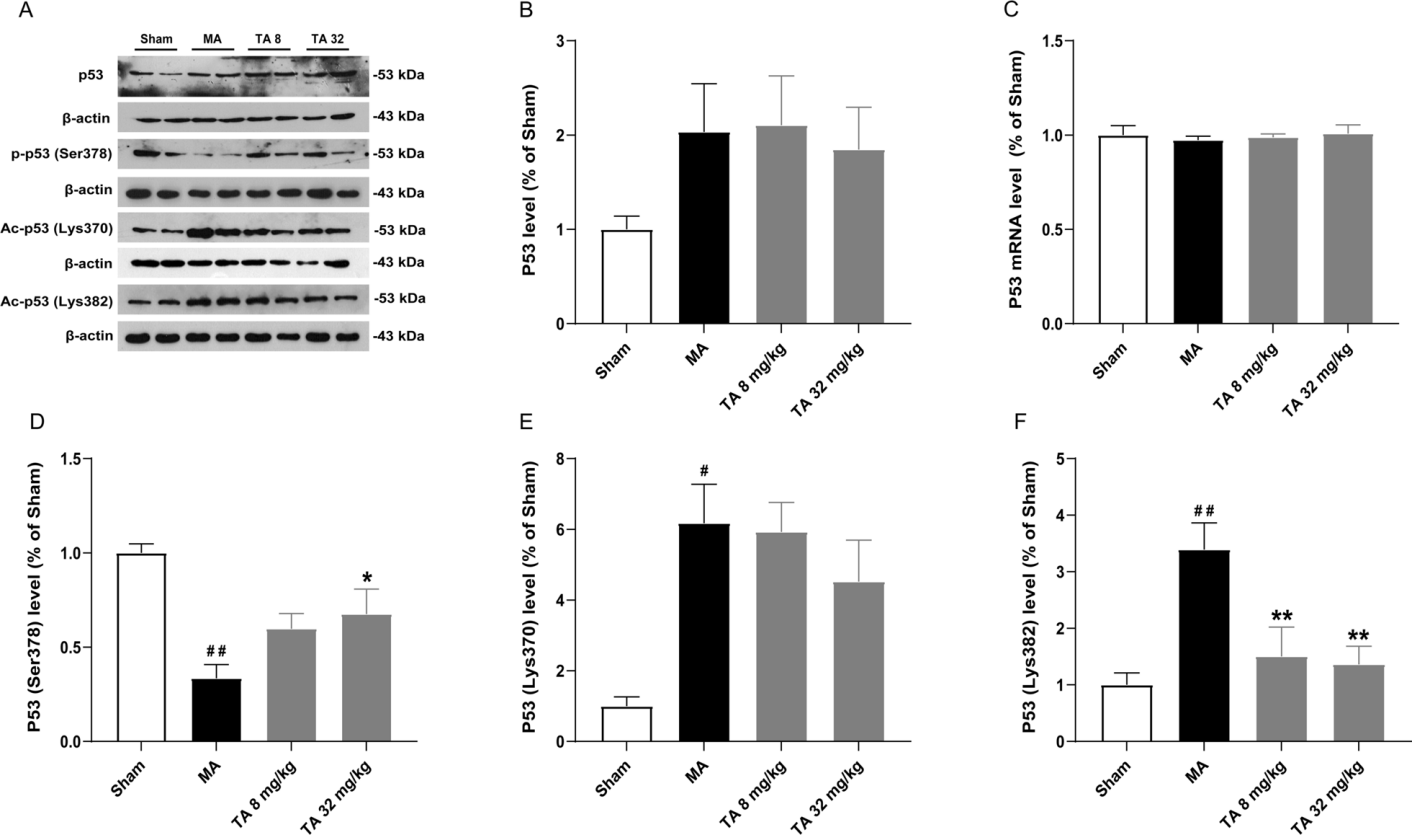
Effect of tolfenamic acid on p53 and the posttranslational modification of p53 in the striatum of malonic acid-injected rats. There was no significant difference in the expression of p53 protein (**A**, **B**) or mRNA (**C**). Malonic acid injection decreased the level of p-p53 Ser378 (**D**) and increased the levels of Ac-p53 Lys370 (**E**) and Ac-p53 Lys382 (**F**). Tolfenamic acid prevented the malonic acid-induced changes in p-p53 Ser378 and Ac-p53 Lys382. All results are expressed as the mean ± SEM. WB: *n* = 5; mRNA: *n* = 3. #*p* < 0.05, ##*p* < 0.01 vs. sham; *p < 0.05, **p < 0.01 vs. MA

It has been reported that the nuclear expression and transcriptional activity of p53 are upregulated in HD transgenic mice and HD patient brains, and knockout of p53 prevents motor deficits in HD transgenic mice. Therefore, we tested the effect of tolfenamic acid on the activity of p53. There was no significant difference in the expression of p53 protein and mRNA levels (The primers could be found in Table 1) in the striatum of rats among all groups (*p* > 0.05, Fig. 4A–C). Next, we tested the effect of tolfenamic acid on the posttranslational modification of p53. Compared with those of the sham group, the phosphorylation level of p53 at the Ser378 site significantly decreased, and the acetylation level of p53 at the Lys370 and Lys382 sites significantly increased in the striatum of the model group rats (*p* < 0.05, Fig. 4A, D–F). Compared with the malonic acid injection group, tolfenamic acid significantly increased the phosphorylation level of p53 at Ser378 and reduced the acetylation level of p53 at Lys382 (*p* < 0.05, Fig. 4).

### Tolfenamic acid regulated genes involved in the p53 pathway in the striatum of the malonic acid-injected rats

**Fig. 5:**
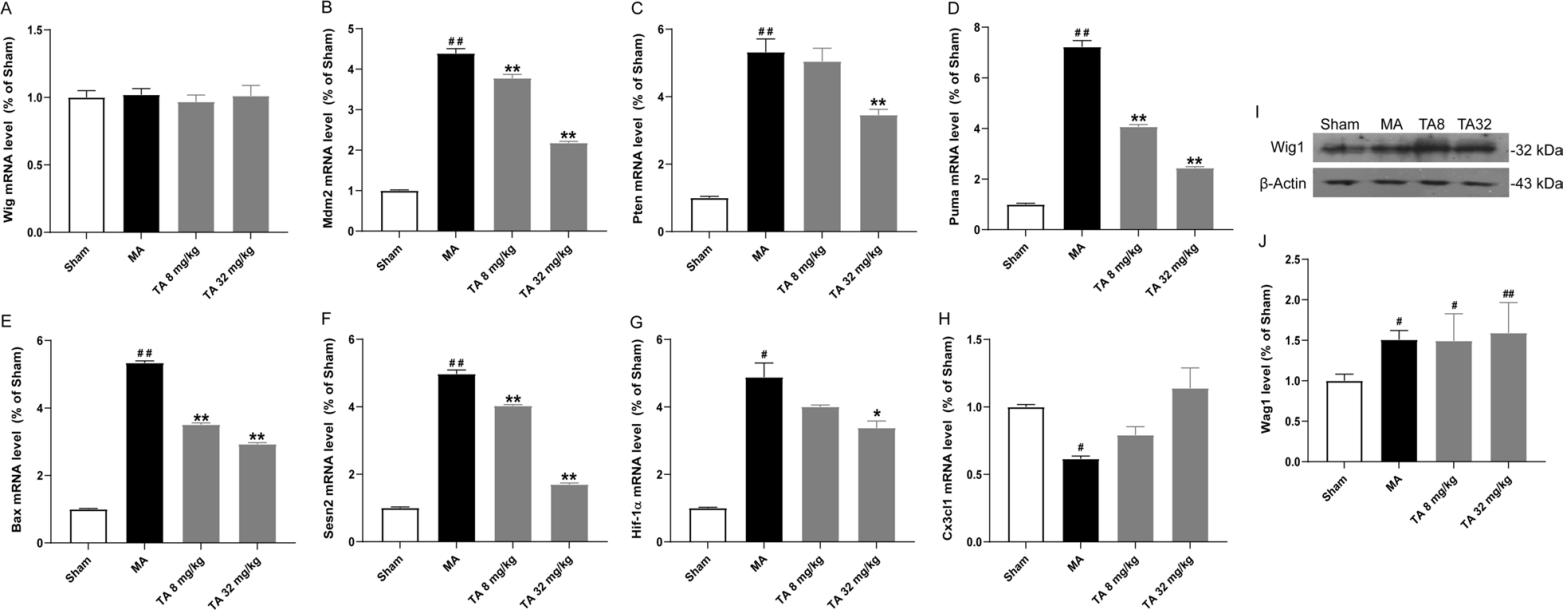
Effect of tolfenamic acid on genes involved in the p53 pathway in the striatum of the malonic acid-injected rats. There was no significant difference in the mRNA level of Wig1 (**A**) among all groups. Tolfenamic acid prevented the malonic acid-induced increases in the protein level of Wig1 and the mRNA levels of Mdm2 (**B**), Pten (**C**), Puma (**D**), Bax (**E**), Sesn2 (**F**) and Hif-1α (**G**) and the decrease in Cx3cl1 (H) mRNA levels. However, compared with the malonic acid-injected rats, both doses of tolfenamic acid could not decrease the protein level of Wig1 (**I**, **J**). All of the results are expressed as the means ± SEMs. PCR: *n* = 3; WB: *n* = 4. ^#^*p* < 0.05, ^##^*p* < 0.01 vs. sham; *p < 0.05, **p < 0.01 vs. MA

**Fig. 6:**
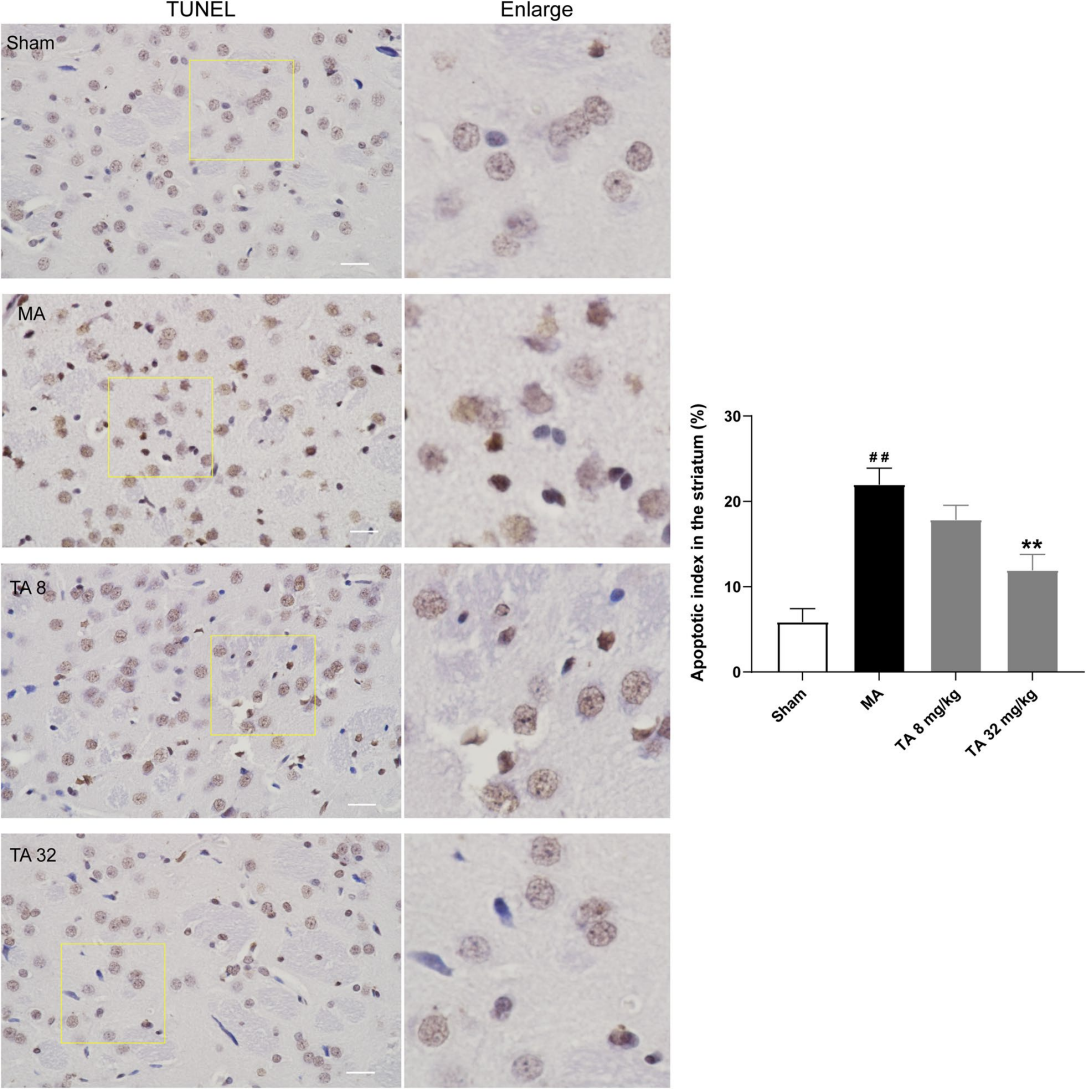
Tolfenamic acid decreased the number of TUNEL-positive cells in the striatum of the malonic acid-treated rats. All of the results are expressed as the means ± SDs. *n* = 4. Scale bars = 250 μm. ^##^*p* < 0.01 vs. sham; **p < 0.01 vs. MA

p53 plays an important role in the regulation of apoptosis. Therefore, we tested the effect of tolfenamic acid on p53-related apoptosis targets. It has been reported that the p53/Wig1 pathway is activated in the brains of HD transgenic mice. However, similar to the results of p53, there was no significant difference in the mRNA level of Wig1 among all groups (*p* > 0.05, Fig. 5A). Malonic acid injection significantly increased the protein expression of Wig1. However, compared with the malonic acid-injected rats, both doses of tolfenamic acid could not decrease the protein level of Wig1 (*p* > 0.05, Fig. 5I, J). Thus, this represents the difference in the gene levels between malonic acid-injected rats and mHtt transgenic mice. Compared with levels in the sham group, the mRNA levels of mouse double minute 2 (Mdm2), phosphatase and tensin homologue (Pten), P53-upregulated modulator of apoptosis (Puma) and Bcl2-associated X (Bax) in the striatum of the malonic acid-injected rats were significantly increased (*p* < 0.05, Fig. 5B–E). Tolfenamic acid (32 mg/kg) obviously decreased the mRNA levels of Mdm2, Pten, Puma and Bax (*p* < 0.01, Fig. 5B–E). DNA fragmentation occurs during apoptosis. Malonic acid injection significantly increased the number of TUNEL-positive cells in the striatum (*p* < 0.01, Fig. 6). Tolfenamic acid decreased the positive cellular rate from 11.91 to 7.47% (*p* < 0.01, Fig. 6). The dose of tolfenamic acid (32 mg/kg) was based on the conversion of a regular dose for mice (50 mg/kg) in our previous study [3] and that reported by Adwan L et al. [19]. At a dose of 50 mg/kg, tolfenamic acid can induce apoptosis in cancer cells of nude mice [20, 21]. However, the total dose of tolfenamic acid that gets through the blood–brain barrier is far less than 50 mg/kg. In an in vitro test, tolfenamic acid induced cancer cell death at 50 μM [20, 21] and showed a neuroprotective effect at 10 μM [3, 19]. Therefore, it is possible that tolfenamic acid has a neuroprotective effect at low doses.

Sestrin 2 (Sesn2) is regulated via stress-responsive transcription factors, including p53, Nrf2, and hypoxia inducible factor 1, alpha subunit (Hif-1α). Compared with levels in the sham group, the mRNA levels of Sesn2 and HIF-1α were significantly increased in the striatum of the model group rats (*p* < 0.01, Fig. 5F, G). Tolfenamic acid (32 mg/kg) obviously decreased the mRNA levels of Sesn2 and HIF-1α (*p* < 0.01, Fig. 5F, G). This result suggested that tolfenamic acid could improve oxidative stress injury in the brains of malonic acid-injected rats. Chemokine C–X3–C motif ligand 1 (Cx3cl1) is a transmembrane chemokine expressed by neurons. Cx3cl1 activates its unique receptor, Cx3cr1, which is expressed in microglia. Activated Cx3cl1 signalling shows neuroprotective effects and prevents neuronal loss in Alzheimer’s disease. Compared with that in the sham group, the Cx3cl1 mRNA level in the striatum of the malonic acid-injected group was significantly decreased (*p* < 0.01, Fig. 5H). Tolfenamic acid increased the Cx3cl1 mRNA level, but the increase was not significant (*p* < 0.01, Fig. 5H).

**Fig. 7:**
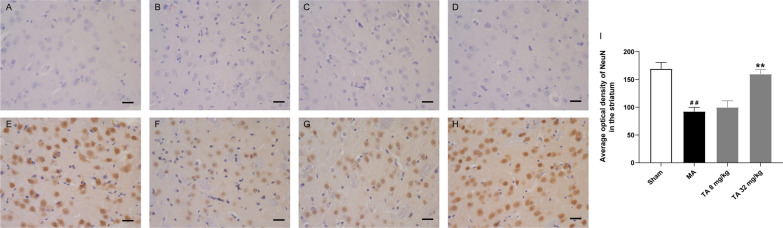
Tolfenamic acid improved cell morphology, as shown by HE staining **A** sham group, **B** MA group, **C** 8 mg/kg TA group, **D** 32 mg/kg TA group), and significantly increased the expression of NeuN (**E** sham group, **F** MA group, **G** 8 mg/kg TA group, **H** 32 mg/kg TA group, **I** quantification of the NeuN level) in the striatum of the malonic acid-injected rats. All of the results are expressed as the means ± SEMs. *n* = 4. Scale bars = 250 μm. ^##^*p* < 0.01 vs. sham; **p < 0.01 vs. MA

Striatal neuron survival plays a vital role in motor and cognitive function. Malonic acid shows obvious toxicity to brain mitochondria. As shown in Fig. 7A. The morphology of the striatum in the sham group was normal. The morphology was shrunken and pyknotic, and the number of cells was decreased in the striatum of the malonic acid-injected group (Fig. 7B). Tolfenamic acid (32 mg/kg) attenuated malonic acid-induced cell injury (Fig. 7C, D). We also used neuronal nuclear antigen (NeuN), a neuronal marker, to test cell injury in the striatum. The expression of NeuN was significantly decreased in the malonic acid-injected rats. Tolfenamic acid (32 mg/kg) prevented this decrease (*p* < 0.05, Fig. 7E–I).

## Discussion

Malonic acid is a mitochondrial SDH inhibitor that can induce mitochondrial dysfunction in mouse brain cells [4]. Intrastriatal injections of malonic acid could induce some pathologies similar to those seen in HD patients, such as striatal neuronal loss, ATP depletion, a significant reduction in cytochrome oxidase activity, ROS production, and mitochondrial swelling in rats [22–24]. The potential sources of ROS in the brain include mitochondrial electron transport and NADPH oxidase. As an isoform of NADPH oxidase, Nox1 contributes to cell growth, angiogenesis, and motility [25]. Nox1-induced ROS in the prefrontal cortex downregulated the level of BDNF, promoting depression in mice [11]. A Nox1 inhibitor prevented methamphetamine-induced blood–brain barrier dysfunction [26]. Nox1 knockout significantly decreased the infarct size and cerebral oedema in middle cerebral artery filament occlusion mice [27]. Tolfenamic acid is a nonsteroidal anti-inflammatory drug and belongs to the fenamates group. It has good antipyretic, analgesic and anti-inflammatory effects. It is clinically used to treat acute migraine, dysmenorrhea, rheumatoid arthritis and other diseases [1]. In our previous study, tolfenamic acid decreased O_2_-levels in 3-nitropropionic acid-treated PC12 cells and activated the Nrf2 pathway in R6/1 HD transgenic mice [3]. In this study, tolfenamic acid significantly decreased the expression of Nox1, lipid peroxidation levels, and the NO and H_2_O_2_ contents and restored CAT and SOD enzyme activities and GSH levels. Thus, tolfenamic acid could prevent Nox1-mediated oxidative stress in malonic acid-injected rats, which supported the antioxidant effect of tolfenamic acid.

Nox1-dependent ROS promote p53 pathway activation and further participate in the cell senescence process [12]. Under physiological conditions, intracellular p53 is maintained at a low level. When cells are stressed, the expression and activity of p53 significantly increase, resulting in cell cycle arrest and apoptosis [13]. In this study, we did not observe changes in p53 at the protein and mRNA levels. That is, there may be no mutant Htt in malonic acid-injected rats, which combines with the p53 promoter to regulate its expression. Another key regulator of p53 transcriptional activity occurs at the posttranslational modification level. p53 can be acetylated at the Lys 370, 372 and 382 residues at its C-terminus in damaged cells [28]. Waterman et al. showed that the Ser376 and 378 sites of the p53 protein were phosphorylated under physiological conditions. p53 is dephosphorylated to increase its activity under stress conditions [29]. In this study, tolfenamic acid significantly increased the phosphorylation level of p53 at the Ser378 site and reduced the acetylation level at the Lys382 site, suggesting that tolfenamic acid may reduce the activity of p53 by posttranslational modification.

Schilling et al. reported that p53 knockout could ameliorate pathological and behavioural deficits in HD transgenic mice [30]. Kim et al. tested 113 genes involved in the p53 pathway in the brains of HD transgenic mice and found that 8 key genes were upregulated more than 1.5-fold compared with those in WT mice. The promoters of all 8 genes, namely, Bax, Puma, Wig1, Sesn2, Mdm2, Hif-1α, Cx3cl1 and Pten, contain p53 binding sites. In this study, we tested whether intrastriatal injections of malonic acid in rats could simulate the above changes in HD transgenic mice and evaluated the effect of tolfenamic acid on genes involved in the p53 pathway.

Mdm2 is a negative regulator of p53. It can directly combine with p53 to form the Mdm2–p53 complex and downregulate p53 activity. Mdm2 overexpression is one of the mechanisms for p53 inactivation [31]. Mdm2′s functional single nucleotide polymorphism rs2279744 (SNP309) can increase the affinity between transcription factor Sp1 and the Mdm2 gene promoter, increase the expression of Mdm2, and further inhibit the activity of p53 [32]. Pten blocks the nuclear translocation of Mdm2 by inhibiting the PI3K/Akt pathway. Decreasing Mdm2 entry into the nucleus will promote the p53 response to apoptosis [33]. To our surprise, in this study, tolfenamic acid, as an Sp1 inhibitor, significantly reduced, but did not increase, the mRNA levels of Mdm2 and Pten. We hypothesized that tolfenamic acid inhibited the activity of p53 by posttranslational modification, therefore, reducing the demand for Mdm2-mediated E3 ubiquitin ligase degradation of p53.

Puma is the downstream target of p53. p53 can induce Puma to form a complex with Bcl-xl [34]. Bax is a pro-apoptotic protein and a member of the Bcl-2 protein family. The Bax gene can be directly activated by the p53 binding site [35]. Wig1, a response gene of p53 [36], is upregulated in the brains of HD patients and HD transgenic mice [15]. Wig1 interacts with Htt mRNA, preferentially with mutant Htt, and mediates HD-associated pathological cellular phenotypes. In this study, tolfenamic acid significantly inhibited the mRNA levels of Puma and Bax. However, no significant difference was found in the Wig1 mRNA levels among all groups, and tolfenamic acid could not decrease the protein level of Wig1. Thus, there was a difference in gene levels between malonic acid-injected rats and mHtt transgenic mice.

Malonic acid disturbs the activity of the oxidative phosphorylase complex; that is, malonic acid induces neurotoxicity, including not only mitochondrial dysfunction and apoptosis but also oxidative stress [17]. We observed that after administration of malonic acid, the mRNA levels of Sesn2, a highly conserved antioxidant protein, and Hif-1α were significantly increased, which may be a compensatory response to fight against oxidative stress injury in the brain. Tolfenamic acid improved the oxidative damage in the brain and restored the mRNA levels of Sesn2 and Hif-1α to normal. Malonic acid activates microglia [37]. The chemokine Cx3cl1 shows a neuroprotective effect on microglia and coordinates motor function [38]. Our results showed that the mRNA level of Cx3cl1 significantly decreased after malonic acid injection. This decrease could be prevented by tolfenamic acid.

Malonic acid is a succinate dehydrogenase inhibitor that shows obvious mitochondrial toxicity. More experiments are needed to further explore the effect of tolfenamic acid on mitochondrial function, including its effect on mitochondrial dynamics and biosynthesis. We should also test the effect of tolfenamic acid on the p53 pathway in mHtt transgenic mice. These are the limitations of our study and represent the future perspectives.

## Conclusions

P53 is activated by posttranslational modification in the brain of intrastriatal malonic acid-injected rats, and tolfenamic acid can inhibit the ROS-generating oxidase Nox1-regulated p53 and its related targets to show a protective effect on malonic acid-induced neurotoxicity.

## Data Availability

The data used to support the findings of this study are available from the corresponding author upon request.

## Abbreviations

Cx3cl1: Chemokine C-X3-C motif ligand 1
HD: Huntington’s disease
Hif-1α: Hypoxia inducible Factor 1, alpha subunit
Mdm2: Mouse double minute 2
mHtt: Mutant huntingtin protein
NeuN: Neuronal nuclear antigen
Nox1: Nicotinamide adenine dinucleotide 3-phosphate (NADPH) oxidase 1
Pten: Phosphatase and tensin homolog
Puma: P53-upregulated modulator of apoptosis
ROS: Reactive oxygen species
Sesn2: Sestrin 2
Sp1: Specificity protein 1
Wig1: Wild-type p53-induced gene 1
